# Comparative effectiveness of nafcillin or cefazolin versus vancomycin in methicillin-susceptible *Staphylococcus aureus *bacteremia

**DOI:** 10.1186/1471-2334-11-279

**Published:** 2011-10-19

**Authors:** Marin L Schweizer, Jon P Furuno, Anthony D Harris, J Kristie Johnson, Michelle D Shardell, Jessina C McGregor, Kerri A Thom, Sara E Cosgrove, George Sakoulas, Eli N Perencevich

**Affiliations:** 1 Department of Epidemiology and Preventive Medicine, University of Maryland School of Medicine, Baltimore, MD, USA; 2 Department of Internal Medicine, University of Iowa Carver College of Medicine, Iowa City, IA, USA; 3 Iowa City VA Health Care System, Iowa City, IA, USA; 4 Department of Pathology, University of Maryland School of Medicine, Baltimore, MD, USA; 5 Department of Pharmacy Practice, College of Pharmacy, Oregon Health & Science University, Portland, OR, USA; 6 Division of Infectious Diseases, Johns Hopkins University School of Medicine, Baltimore, MD, USA; 7 Department of Pediatrics, University of San Diego School of Medicine, La Jolla, CA; 8 Department of Medicine, Sharp Memorial Hospital, San Diego, CA

## Abstract

**Background:**

The high prevalence of methicillin-resistant *S. aureus *(MRSA) has led clinicians to select antibiotics that have coverage against MRSA, usually vancomycin, for empiric therapy for suspected staphylococcal infections. Clinicians often continue vancomycin started empirically even when methicillin-susceptible *S. aureus *(MSSA) strains are identified by culture. However, vancomycin has been associated with poor outcomes such as nephrotoxicity, persistent bacteremia and treatment failure. The objective of this study was to compare the effectiveness of vancomycin versus the beta-lactam antibiotics nafcillin and cefazolin among patients with MSSA bacteremia. The outcome of interest for this study was 30-day in-hospital mortality.

**Methods:**

This retrospective cohort study included all adult in-patients admitted to a tertiary-care facility between January 1, 2003 and June 30, 2007 who had a positive blood culture for MSSA and received nafcillin, cefazolin or vancomycin. Cox proportional hazard models were used to assess independent mortality hazards comparing nafcillin or cefazolin versus vancomycin. Similar methods were used to estimate the survival benefits of switching from vancomycin to nafcillin or cefazolin versus leaving patients on vancomycin. Each model included statistical adjustment using propensity scores which contained variables associated with an increased propensity to receive vancomycin.

**Results:**

267 patients were included; 14% (38/267) received nafcillin or cefazolin, 51% (135/267) received both vancomycin and either nafcillin or cefazolin, and 35% (94/267) received vancomycin. Thirty (11%) died within 30 days. Those receiving nafcillin or cefazolin had 79% lower mortality hazards compared with those who received vancomycin alone (adjusted hazard ratio (HR): 0.21; 95% confidence interval (CI): 0.09, 0.47). Among the 122 patients who initially received vancomycin empirically, those who were switched to nafcillin or cefazolin (66/122) had 69% lower mortality hazards (adjusted HR: 0.31; 95% CI: 0.10, 0.95) compared to those who remained on vancomycin.

**Conclusions:**

Receipt of nafcillin or cefazolin was protective against mortality compared to vancomycin even when therapy was altered after culture results identified MSSA. Convenience of vancomycin dosing may not outweigh the potential benefits of nafcillin or cefazolin in the treatment of MSSA bacteremia.

## Background

Through the American Recovery and Reinvestment Act of 2009, Congress requested that the Institute of Medicine (IOM) recommend national priorities for research questions to be addressed by Comparative Effectiveness Research. The first quartile of the IOM's list included a call to compare the effectiveness of strategies for reducing healthcare-associated infections [1]. One such strategy to reduce antibiotic resistant healthcare-associated infections would be to optimize antibiotic therapy.

The high prevalence of methicillin-resistant *Staphylococcus aureus *(MRSA) has led clinicians to select antibiotics that have coverage against MRSA, usually vancomycin, for empiric therapy for suspected staphylococcal infections [2,3] Patients treated initially with empiric vancomycin are frequently continued on vancomycin for dosing convenience even after culture results identify methicillin-susceptible *S. aureus *(MSSA), particularly in patients with renal insufficiency [4]. However, high vancomycin selective pressure may lead to decreased susceptibility to vancomycin in both MSSA and MRSA [5].

Vancomycin has been associated with poor outcomes such as nephrotoxicity, persistent bacteremia and treatment failure among MSSA patients [3,5-9]. Higher mortality in patients with MRSA bacteremia compared to patients with MSSA bacteremia has been attributable to differences in host viability, differences in microbial pathogenicity, and differences in antimicrobial potency, particularly the inferior anti-staphylococcal killing of glycopeptides when compared to the beta-lactam antibiotics nafcillin or cefazolin [7,8,10,11].

Comparative effectiveness research methods are an ideal methodology to compare treatments for *S. aureus *infections because these methods aim to determine effectiveness in the real-world setting. This study aimed to assess differences in 30-day in-hospital mortality in patients with MSSA bacteremia treated with nafcillin or cefazolin compared to those treated with vancomycin. Additionally, we assessed whether those treated empirically with vancomycin would benefit if switched to nafcillin or cefazolin once MSSA was microbiologically identified.

## Methods

### Study design and patient population

This retrospective cohort study included adult patients admitted to the University of Maryland Medical Center, a 656-bed tertiary care facility, between January 1, 2003 and June 30, 2007. Patients were included in the study if they had a positive blood culture for MSSA and received vancomycin, nafcillin or cefazolin. Patients with polymicrobial infections were excluded in order to only assess the antibiotics and outcomes associated with *S. aureus *infection. Patients who only received antibiotics other than vancomycin, nafcillin, or cefazolin were excluded from the study. However, if a patient received vancomycin, nafcillin or cefazolin and another antibiotic (e.g. vancomycin and piperacillin/tazobactam) they were included in the analysis.

Each admission was handled as an independent event and therefore patients could be included in the study more than once. Eligible patients were identified using a relational database that contains medical, pharmaceutical and microbiologic data. These data have been validated in previous studies and have positive and negative predictive values in excess of 99 percent when compared to paper medical records [12,13]. Additional variables (e.g. components of the modified Acute Physiology Score [APS]) that were not available in the relational database were collected by a research nurse via chart review. This study was approved by the institutional review board of the University of Maryland, Baltimore.

### Variable definitions

The primary outcome of interest was 30-day in-hospital mortality, which was defined as mortality occurring during the index hospital admission in the time period from culture collection to 30 days after culture collection. The outcome was censored if the patient survived longer than 30 days after culture collection or if the patient was discharged alive within 30 days after culture collection.

Severity of illness was measured 24 hours before the time the culture was obtained using the modified APS. If the blood culture was obtained within 24 hours of hospital admission, APS at the time of admission was calculated. The modified APS is based on the Acute Physiology and Chronic Health Evaluation (APACHE) III score [14-16]. Since the APACHE III was designed for use among intensive care unit (ICU) patients, the score has been modified by excluding variables that are not applicable to this study population [12-17]. The modified APS includes age, chronic health (AIDS, hepatic failure, lymphoma, metastatic cancer, leukemia/multiple myeloma, immunosupression, cirrhosis), and acute physiologic abnormalities including pulse rate, mean blood pressure, temperature, respiratory rate, hematocrit, white blood cell count, creatinine, pH, blood urea nitrogen, sodium, albumin, bilirubin, and glucose [12-17]. The Charlson Comorbidity Index, an aggregate comorbidity score, was calculated using discharge *International Classification of Diseases, 9^th ^Revision, Clinical Modification *(*ICD-9-CM*) codes for comorbid conditions [18]. Blood cultures collected within 48 hours of admission were designated as community-associated infections. Time to receipt of nafcillin or cefazolin was measured from the time the blood culture was collected to the time the patient first received nafcillin or cefazolin. Similarly, time to receipt of appropriate therapy was measured from the time the blood culture was collected to the time the patient first received an antibiotic in which the *S. aureus *isolate from the blood culture was susceptible *in vitro *[19].

Antimicrobial susceptibility profiles were determined according to Clinical and Laboratory Standards Institute guidelines [20]. Endocarditis was defined as presence of an *ICD-9-CM *code for endocarditis or echocardiographic findings of vegetation on the index admission. Hemodialysis was defined as presence of an *ICD-9-CM *code for hemodialysis or chronic renal disease on the index admission. Pneumonia and osteomyelitis were defined by *ICD-9-CM *codes on the index admission. Patients with a positive wound culture before the first positive blood culture were classified as having a wound infection.

### Statistical analysis

Bivariate associations were assessed using the chi-square test or Fisher's exact test for categorical variables and the Students t-test or the Wilcoxon Rank Sum test for continuous variables. Logistic regression models were used to create two different propensity scores. First, a propensity score was created using variables that were independently associated with receipt of nafcillin or cefazolin versus vancomycin. A separate second propensity score was created using variables that were independently associated with switching from vancomycin to nafcillin or cefazolin versus remaining on vancomycin for use in the model assessing the clinical benefits of switching to nafcillin or cefazolin if started on vancomycin.

Cox proportional hazard models were then fit to measure hazard ratios (HRs) and 95% confidence intervals (CIs) for the associations of interest. The first Cox proportional hazard model assessed the association between receipt of nafcillin or cefazolin versus vancomycin and mortality controlling for the first propensity score. The second Cox proportional hazard model assessed the association between switching from vancomycin to nafcillin or cefazolin versus remaining on vancomycin and mortality controlling for the second propensity score. All analyses were performed using SAS software (SAS Institute, Cary, NC) version 9.1.

## Results

Overall, 326 patients had MSSA bacteremia during the study period. Thirty-one patients were excluded from the analysis because they did not receive nafcillin, cefazolin or vancomycin on the index admission. Of the 31 excluded patients, 14 (45%) did not receive anti-staphylococcal antibiotic therapy and 17 (55%) did not receive vancomycin, nafcillin or cefazolin but received other anti-staphylococcal antibiotics. Twenty-eight patients with polymicrobial infections were excluded. A total of 267 hospital admissions from 252 individual patients were included in the final analysis. Fourteen percent (38/267) received nafcillin or cefazolin alone, 51% (135/267) received both vancomycin and either nafcillin or cefazolin and 35% (94/267) received vancomycin alone. Thirty (11%) patients died within 30 days of culture collection.

All patients in this cohort received vancomycin, nafcillin, or cefazolin. These patients also could have received other antibiotics. During the index admission, 40% of the cohort received piperacillin/tazobactam, 20% received a third-generation cephalosporin, 12% received trimethoprim/sulfamethoxazole, 12% received ampicillin/sulbactam, 10% received clindamycin, 9% received cefepime, 7% received imipenem, 6% received linezolid, 2% received daptomycin, and 2% received a second-generation cephalosporin. Patients who received nafcillin or cefazolin but not vancomycin were significantly less likely to receive other anti-staphylococcal antibiotics compared to patients who received vancomycin (p < 0.01).

Nine percent of patients had a transthoracic echocardiogram (TTE) then a transesophageal echocardiography (TEE) performed, 30% had only a TTE and 5% had only a TEE performed. Of the 40 patients classified as having endocarditis, 60% were classified by echocardiographic findings of vegetation and 40% were classified via an *ICD-9-CM *code for endocarditis.

Table 1 displays the association between patient and treatment factors and receipt of antibiotics. Patients who received nafcillin or cefazolin were less likely to have renal disease and less likely to undergo hemodialysis, were less likely to have a central venous catheter, and had lower median severity of illness and comorbidity scores compared to patients who received vancomycin (p < 0.05). Patients who received nafcillin or cefazolin were more likely to have endocarditis (p < 0.01). Patients who received nafcillin or cefazolin were not significantly different than those who received vancomycin in regards to diagnosis of pneumonia, osteomyelitis, or wound infections. Patients receiving nafcillin or cefazolin were somewhat less likely to have their central venous catheter removed (p = 0.08). However, removal of a central venous catheter was not associated with mortality (p = 0.83) and it was not a confounder in the association between receipt of cefazolin or nafcillin and mortality.

**Table 1:** Characteristics of the study population stratified by receipt of nafcillin or cefazolin

	Received only nafcillin or cefazolin (n = 38)	Received both nafcillin or cefazolin and vancomycin (n = 135)	Received only vancomycin (n = 94)	*p*
**Diabetes**	5 (13.2%)	7 (5.2%)	13 (14.0%)	0.06

**Renal disease**	0 (0%)	9 (6.7%)	13 (14.0%)	0.02

**Severity of illness score, median, IQR**	11.5 (6.0, 16.0)	16.0 (7.0, 25.0)	21.0 (14.0, 29.0)	< 0.01

**Charlson Comorbidity Index, median, IQR**	0 (0, 2)	1 (0, 3)	2 (1,4)	< 0.01

**Endocarditis**	9 (23.7%)	28 (20.7%)	3 (3.2%)	< 0.01

**Pneumonia**	1 (2.6%)	12 (8.9%)	3 (3.2%)	0.13

**Osteomyelitis**	1 (2.6%)	7 (5.2)	1 (1.1%)	0.22

**Wound Infection**	3 (7.9%)	4 (3.0%)	2 (2.1%)	0.23

**Hemodialysis**	0 (0%)	13 (9.6%)	18 (19.2%)	< 0.01

**Community-associated infection**	26 (68.4%)	94 (69.6%)	54 (57.5%)	0.14

**ICU admission before culture collection**	6 (15.8%)	40 (29.6%)	28 (29.8%)	0.20

**Age, mean ± SD, year**	51.0 ± 16.4	48.0 ± 15.6	49.8 ± 16.9	0.78

**Female sex**	17 (44.7%)	48 (35.6%)	32 (34.0%)	0.49

**Presence of central venous catheter**	19 (50.0%)	61 (45.2%)	59 (62.8%)	0.03

**Central venous catheter removed**	8/19 (42.1%)	43/61 (70.5%)	38/59 (64.4%)	0.08

Data are no. (%) of admissions, unless otherwise indicated. IQR, interquartile range; ICU, intensive care unit; SD, standard deviation.

When 30-day in-hospital mortality was stratified by antibiotics received during the index admission, we found cumulative mortality incidences of 3% (1/38) among patients who received only nafcillin or cefazolin, 7% (10/135) among patients who received both vancomycin and nafcillin or vancomycin and cefazolin, and 20% (19/94) among patients who only received vancomycin (chi-square test for trend p < 0.01). When we excluded patients who received both vancomycin and nafcillin or vancomycin and cefazolin, those who received nafcillin or cefazolin only were less likely to die compared to those who received vancomycin only (unadjusted HR: 0.10; 95% CI: 0.01, 0.76). Those who received nafcillin or cefazolin, including those who received both vancomycin and nafcillin or cefazolin, were significantly less likely to die compared to patients who received only vancomycin, after statistically adjusting for the propensity score which included severity of illness, aggregate comorbidity, age, admission to the ICU prior to culture collection, hemodialysis, endocarditis, time to receipt of appropriate therapy, and receipt of other anti-staphylococcal antibiotics (adjusted HR: 0.21; 95% CI: 0.09, 0.47) (Table 2).

**Table 2:** Propensity score adjusted associations between receipt of nafcillin or cefazolin versus vancomycin and 30-day in-hospital mortality

Association	Adjusted hazard ratio and 95% confidence interval	Variables included in the propensity score
Receipt of nafcillin or cefazolin versus vancomycin and 30-day in-hospital mortality	**0.21 (0.09, 0.47)**	**Severity of illness** **Aggregate comorbidity** **Admission to the ICU** **Age** **Hemodialysis** **Endocarditis** **Time to receipt of appropriate therapy** **Receipt of other anti** **-staphylococcal antibiotic**

Switch from vancomycin to nafcillin or cefazolin versus remaining on vancomycin and 30-day in-hospital mortality	**0.31 (0.10, 0.95)**	**Severity of illness** **Aggregate comorbidity** **Admission to the ICU** **Community-associated infection** **Hemodialysis** **Age** **Endocarditis** **Time to receipt of appropriate therapy**

In order to evaluate the benefit of switching therapy from vancomycin to nafcillin or cefazolin, we examined the cohort of patients who received vancomycin empirically, excluding the 13 patients who received nafcillin or cefazolin before receipt of vancomycin. Of the 122 patients in this sub-cohort, 66 (54%) were switched from vancomycin to nafcillin or cefazolin. The median time from receipt of vancomycin to the switch to nafcillin or cefazolin was 3.0 days (interquartile range: 2.4, 3.9 days).

Patients who switched from vancomycin to nafcillin or cefazolin were less likely to die compared to those who remained on vancomycin (unadjusted HR: 0.33; 95% CI: 0.13, 0.87). This association remained significant after statistically adjusting for the propensity score which included severity of illness, aggregate comorbidity, admission to the ICU prior to culture collection, community-associated infection, time to receipt of appropriate therapy, endocarditis, hemodialysis, and age (adjusted HR: 0.31; 95% CI: 0.10, 0.95) (Table 2). The association remained when the six patients who died or were discharged within 24 hours of culture collection were excluded. Similarly, switching from vancomycin to nafcillin or cefazolin remained protective when this variable was treated as a time varying covariate in the statistical analysis.

Among the subset of 139 patients who had a central venous catheter, receipt of nafcillin or cefazolin was protective against mortality after statistically adjusting for the propensity score (adjusted HR: 0.26; 95% CI: 0.10, 0.69). When the cohort was stratified by severity of illness (modified APS≤16 or > 16), the cumulative mortality incidences in both strata were lowest among patients who received nafcillin or cefazolin only (0% and 11.1% respectively), followed by patients who received both vancomycin and nafcillin or cefazolin (1.4% and 13.9% respectively) and highest among patients who received vancomycin only (8.6% and 27.1% respectively).

When only the first admission per patient was analyzed, the results did not change. Receipt of nafcillin and cefazolin at anytime (adjusted HR: 0.20; 95% CI: 0.09, 0.44) and switching from vancomycin to nafcillin or cefazolin (adjusted HR: 0.28; 95% CI: 0.09, 0.86) remained protective against mortality.

When analyzed separately, both nafcillin and cefazolin were protective against mortality. These associations remained after adjusting for propensity scores, although the association between nafcillin and mortality was not statistically significant (nafcillin adjusted HR: 0.45; 95% CI: 0.18, 1.15; cefazolin adjusted HR: 0.25; 95% CI: 0.09, 0.66). Additionally, among the 173 patients who ever received nafcillin or cefazolin, 6% died within 30 days of culture collection. Patients who died had a longer time to receipt of nafcillin or cefazolin (mean = 4.0 days, standard deviation [SD] = 4.5 days) compared to those who survived (mean = 2.5 days, SD = 6.6 days).

## Discussion

Clinicians are often faced with therapeutic trade-offs that balance risk and benefit. When faced with treating staphylococcal infections, this may include balancing the ease of administration of vancomycin compared to the more challenging administration of beta-lactam antibiotics such as nafcillin with a potential trade-off in efficacy. In this comparative effectiveness analysis, we found that receipt of nafcillin or cefazolin in MSSA bacteremic patients was independently associated with a 79% lower adjusted rate of mortality compared to vancomycin. In addition, switching antimicrobial therapy from vancomycin to nafcillin or cefazolin was also highly protective against mortality. Thus, while the initiation of empiric vancomycin therapy in cases of suspected *S. aureus *bacteremia is reasonable pending microbiological confirmation of *S. aureus *and susceptibilities, this data provides strong evidence for the importance of switching therapy to nafcillin or cefazolin once culture results confirm MSSA bacteremia. The benefit was present in this cohort even though the median time to switching was three days, suggesting that it is beneficial to switch away from vancomycin even in settings without access to rapid diagnostics.

This study found that the probability of survival was greater among patients who received only nafcillin or cefazolin compared to those who received both vancomycin and nafcillin or cefazolin. It could be postulated that clinicians may prescribe more antibiotics to the patients with severe underlying conditions, which could lead to these results. However, this trend remained even after the cohort was stratified by severity of illness. Another potential reason for these differences may be due to beta-lactam antagonism, perhaps through penicillin-binding protein substrate competition [21,22]. However, a recent study found a lack of antagonism between oxacillin and vancomycin against MSSA [23].

The differences in mortality among patients who received vancomycin compared to those who received nafcillin or cefazolin may also be explained by pharmacodynamic differences among these agents, particularly with respect to their interactions with innate host defense molecules. Not only are nafcillin or cefazolin more potent bactericidal agents *in vitro *compared to vancomycin, but they also act synergistically with innate host defense cationic antimicrobial peptides such as platelet microbicidal proteins (PMPs) in killing *S. aureus *[24].

The recent clinical practice guidelines by the Infectious Disease Society of America for the treatment of MRSA stated that vancomycin "is clearly inferior to beta-lactams for MSSA bacteremia and infective endocarditis" and cited five studies to support this statement [25]. Two of the cited studies assessed the use of vancomycin among intravenous drug users with endocarditis [26,27]. The three other cited studies compared outcomes among patients with *S. aureus *bacteremia who received vancomycin and those who received nafcillin or cefazolin [7-9]. Chang et al. found that nafcillin was superior to vancomycin in preventing recurrence of *S. aureus *bacteremia but that study was not designed to assess mortality and did not assess the impact of switching from vancomycin to nafcillin [7]. In contrast, Stryjewski et al. evaluated a cohort of hemodialysis-dependent patients with MSSA bacteremia, in which all patients initially received vancomycin. They found that treatment failure was more common among patients who continued on vancomycin compared to those who were switched to a first-generation cephalosporin (adjusted Odds Ratio: 3.5; 95% CI: 1.2, 13.5). In that study, treatment failure was defined as death or recurrent infection, but it was not sufficiently powered to assess mortality alone [8]. Kim et al., compared receipt of vancomycin versus beta-lactams among MSSA bacteremia patients admitted to two South Korean hospitals. They found that receipt of vancomycin was associated with increased mortality (adjusted OR: 3.3; 95% CI: 1.2, 9.5). However, less than 10% of the patients in that study received vancomycin. Thus their results may not be generalizable to other populations in which a higher proportion of *S. aureus *bacteremic patients receive vancomycin [9]. Additionally, three more studies compared vancomycin to beta-lactam antibiotics among patients with endocarditis or pneumonia and found that those who received vancomycin had worse patient outcomes [28-30].

None of the prior studies assessed the benefits of switching from vancomycin to nafcillin or cefazolin in a general patient population, as we have shown here. This finding identifies a potential target for interventions designed to improve antibiotic prescribing and infectious disease outcomes. A clinical alert or reminder to switch to nafcillin or cefazolin in non-allergic patients with MSSA bacteremia would be expected to improve clinical outcomes with minimal costs.

This current study is a subset of a larger cohort of all UMMC patient admissions with *S. aureus *bacteremia, including both MRSA and MSSA. Similar to our current findings, in the larger cohort we found that appropriate definitive antibiotic therapy was protective against mortality; however, appropriate empiric antibiotic therapy was not protective against mortality [19]. Thus, a short time to receipt of antibiotics may not be as important as eventual receipt of optimal antibiotics.

Our study had several limitations. The results could be affected by residual confounding because patients who received nafcillin or cefazolin were healthier compared to those who received vancomycin. However, through our propensity scores we controlled for many factors that may affect health such as severity of illness, aggregate comorbidity, endocarditis and receipt of other anti-staphylococcal antibiotics. In addition, our multivariable analysis suggested that switching to nafcillin or cefazolin was associated with a 69% reduction in risk of 30-day mortality. It would be unlikely that residual confounding, beyond the numerous factors that we already controlled for, could explain away such a profound protective effect. The receipt of other anti-staphylococcal antibiotics may also have led to residual confounding in this study. Yet, patients who received nafcillin or cefazolin were significantly less likely to receive other antibiotics compared to patients who received vancomycin. Additionally, there was no significant difference in mortality between patients who received only vancomycin versus vancomycin plus another antibiotic other than nafcillin or cefazolin (data not shown). Thus, the benefits of additional antibiotics would bias our results toward the null.

Our study was limited by its retrospective observational design. A randomized control study design would be preferred to study this association, but some clinicians may consider it unethical to randomly assign patients with MSSA to vancomycin given these findings. Due to the retrospective design of this study, we were not able to evaluate the duration or drug levels of antibiotic treatment. Thus under-dosing may be unrecognized, especially among hemodialysis patients. Additionally, only 19% of the patients with *S. aureus *bacteremia had an echocardiographic evaluation and only 60% of cases classified as endocarditis had an echocardiography. Thus, our measurement of endocarditis may be less accurate than measurements done prospectively.

This study suggests that patients with MSSA bacteremia should receive nafcillin or cefazolin as soon as the pathogen is definitively identified by culture since there was a 69% lower risk of death in those patients who were switched from vancomycin. Thus, these results imply that clinicians should not continue vancomycin for dosing scheduling convenience, as any benefits from simplified dosing schedules would be greatly outweighed by the survival benefits of switching to nafcillin or cefazolin. An unmeasured potential implication of this study is that the choice to switch patients from vancomycin to nafcillin or cefazolin may decrease vancomycin selection pressure, thus delaying the emergence of decreased susceptibility to vancomycin.

## Conclusions

In summary, receipt of nafcillin or cefazolin was independently associated with decreased mortality compared to vancomycin in MSSA bacteremia therapy. This benefit persists even if receipt is delayed until definitive culture results are available. Choice of empiric antibiotic therapy must weigh MRSA coverage against potentially superior antibiotics. Nafcillin or cefazolin should be considered as the treatment of choice for definitive therapy for MSSA bacteremia.

## Financial and non-financial competing interests

This study was funded in part by an investigator-initiated research grant from Pfizer, Inc (IIR GA5951BG). ML Schweizer had full access to all of the data in the study and takes responsibility for the integrity of the data and the accuracy of the data analysis.

## Authors' contributions

MLS: participated in the design of the study, performed statistical analysis and wrote the manuscript; JPF, ADH, JKJ, MDS, JCM, KAT, SEC, GS, ENP: participated in the design of the study and helped to draft the manuscript. All authors read and approved the final manuscript.

## Pre-publication history

The pre-publication history for this paper can be accessed here:

http://www.biomedcentral.com/1471-2334/11/279/prepub

## References

[B1] Institute of Medicine Initial national priorities for comparative effectiveness research 2009 Washington DC: National Academies Pr

[B2] Johnson JK Khoie T Shurland S Kreisel K Stine OC Roghmann MC Skin and soft tissue infections caused by methicillin-resistant *Staphylococcus aureus *USA300 clone Emerg Infect Dis 2007 13 8 1195 200 17953091 10.3201/eid1308.061575 PMC2828080

[B3] DeLeo FR Chambers HF Reemergence of antibiotic-resistant *Staphylococcus aureus *in the genomics era J Clin Invest 2009 119 9 2464 74 10.1172/JCI38226 19729844 PMC2735934

[B4] Barth RH DeVincenzo N Use of vancomycin in high-flux hemodialysis: Experience with 130 courses of therapy Kidney Int 1996 50 3 929 36 10.1038/ki.1996.393 8872968

[B5] Centers for Disease Control and Prevention (CDC) *Staphylococcus aureus *resistant to vancomycin--United States, 2002 MMWR Morb Mortal Wkly Rep 2002 51 26 565 7 12139181

[B6] Lodise TP Lomaestro B Graves J Drusano GL Larger vancomycin doses (at least four grams per day) are associated with an increased incidence of nephrotoxicity Antimicrob Agents Chemother 2008 52 4 1330 6 10.1128/AAC.01602-07 18227177 PMC2292536

[B7] Chang FY Peacock JE Musher DM Triplett P MacDonald BB Mylotte JM *Staphylococcus aureus *bacteremia: Recurrence and the impact of antibiotic treatment in a prospective multicenter study Medicine (Baltimore) 2003 82 5 333 9 10.1097/01.md.0000091184.93122.09 14530782

[B8] Stryjewski ME Szczech LA Benjamin DK Inrig JK Kanafani ZA Engemann JJ Use of vancomycin or first-generation cephalosporins for the treatment of hemodialysis-dependent patients with methicillin-susceptible *Staphylococcus aureus *bacteremia Clin Infect Dis 2007 44 2 190 6 10.1086/510386 17173215

[B9] Kim SH Kim KH Kim HB Kim NJ Kim EC Oh MD Outcome of vancomycin treatment in patients with methicillin-susceptible *Staphylococcus aureus *bacteremia Antimicrob Agents Chemother 2008 52 1 192 7 10.1128/AAC.00700-07 17984229 PMC2223910

[B10] Cosgrove SE Sakoulas G Perencevich EN Schwaber MJ Karchmer AW Carmeli Y Comparison of mortality associated with methicillin-resistant and methicillin-susceptible *Staphylococcus aureus *bacteremia: A meta-analysis Clin Infect Dis 2003 36 1 53 9 10.1086/345476 12491202

[B11] Marra AR Edmond MB Forbes BA Wenzel RP Bearman GM Time to blood culture positivity as a predictor of clinical outcome of *Staphylococcus aureus *bloodstream infection J Clin Microbiol 2006 44 4 1342 6 10.1128/JCM.44.4.1342-1346.2006 16597860 PMC1448655

[B12] Osih RB McGregor JC Rich SE Moore AC Furuno JP Perencevich EN Impact of empiric antibiotic therapy on outcomes in patients with *Pseudomonas aeruginosa *bacteremia Antimicrob Agents Chemother 2007 51 3 839 44 10.1128/AAC.00901-06 17194829 PMC1803143

[B13] Furuno JP Harris AD Wright MO Hartley DM McGregor JC Gaff HD Value of performing active surveillance cultures on intensive care unit discharge for detection of methicillin-resistant *Staphylococcus aureus* Infect Control Hosp Epidemiol 2007 28 6 666 70 10.1086/518348 17520538

[B14] Knaus WA Wagner DP Draper EA Zimmerman JE Bergner M Bastos PG The APACHE III prognostic system, risk prediction of hospital mortality for critically ill hospitalized adults Chest 1991 100 6 1619 36 10.1378/chest.100.6.1619 1959406

[B15] Mylotte JM Aeschlimann JR Rotella DL *Staphylococcus aureus *bacteremia: Factors predicting hospital mortality Infect Control Hosp Epidemiol 1996 17 3 165 8 10.1086/647264 8708354

[B16] Yzerman EP Boelens HA Tjhie JH Kluytmans JA Mouton JW Verbrugh HA Delta APACHE II for predicting course and outcome of nosocomial *Staphylococcus aureus *bacteremia and its relation to host defense J Infect Dis 1996 173 4 914 9 10.1093/infdis/173.4.914 8603971

[B17] Sunenshine RH Wright MO Maragakis LL Harris AD Song X Hebden J Multidrug-resistant Acinetobacter infection mortality rate and length of hospitalization Emerg Infect Dis 2007 13 1 97 103 10.3201/eid1301.060716 17370521 PMC2725827

[B18] Deyo RA Cherkin DC Ciol MA Adapting a clinical comorbidity index for use with ICD-9-CM administrative databases J Clin Epidemiol 1992 45 6 613 9 10.1016/0895-4356(92)90133-8 1607900

[B19] Schweizer ML Furuno JP Harris AD Johnson JK Shardell MD McGregor JC Empiric antibiotic therapy for *Staphylococcus aureus *bacteremia may not reduce in-hospital mortality: A retrospective cohort study PLoS One 2010 5 7 e11432 10.1371/journal.pone.0011432 20625395 PMC2896397

[B20] Clinical and Laboratory Standards Institute Performance standards for antimicrobial susceptibiliy testing CLSI document 2008 Nineteenth Informational Supplement ed. M100 S19

[B21] Aritaka N Hanaki H Cui L Hiramatsu K Combination effect of vancomycin and beta-lactams against a *Staphylococcus aureus *strain, Mu3, with heterogeneous resistance to vancomycin Antimicrob Agents Chemother 2001 45 4 1292 4 10.1128/AAC.45.4.1292-1294.2001 11257050 PMC90459

[B22] Oshiro T Nagasawa Z Hanaki H Ikeda-Dantsuji Y Nagayama A The antagonistic effects of a combination of vancomycin and minocycline in *Staphylococcus aureus *with heterogeneous resistance to vancomycin J Infect Chemother 2008 14 1 15 22 10.1007/s10156-007-0569-9 18297444

[B23] Joukhadar C Pillai S Wennersten C Moellering RC Eliopoulos GM Lack of bactericidal antagonism or synergism in vitro between oxacillin and vancomycin against methicillin-susceptible strains of *Staphylococcus aureus* Antimicrob Agents Chemother 2010 54 2 773 7 10.1128/AAC.00348-09 19933805 PMC2812142

[B24] Dhawan VK Bayer AS Yeaman MR Thrombin-induced platelet microbicidal protein susceptibility phenotype influences the outcome of oxacillin prophylaxis and therapy of experimental *Staphylococcus aureus *endocarditis Antimicrob Agents Chemother 2000 44 11 3206 9 10.1128/AAC.44.11.3206-3209.2000 11036055 PMC101635

[B25] Liu C Bayer A Cosgrove SE Daum RS Fridkin SK Gorwitz RJ Clinical practice guidelines by the Infectious Diseases Society of America for the treatment of methicillin-resistant *Staphylococcus aureus *infections in adults and children Clin Infect Dis 2011 52 3 e18 55 10.1093/cid/ciq146 21208910

[B26] Small PM Chambers HF Vancomycin for *Staphylococcus aureus *endocarditis in intravenous drug users Antimicrob Agents Chemother 1990 34 6 1227 31 2393284 10.1128/aac.34.6.1227 PMC171789

[B27] Lodise TP McKinnon PS Levine DP Rybak MJ Impact of empirical-therapy selection on outcomes of intravenous drug users with infective endocarditis caused by methicillin-susceptible *Staphylococcus aureus* Antimicrob Agents Chemother 2007 51 10 3731 3 10.1128/AAC.00101-07 17664322 PMC2043293

[B28] Gentry CA Rodvold KA Novak RM Hershow RC Naderer OJ Retrospective evaluation of therapies for *Staphylococcus aureus *endocarditis Pharmacotherapy 1997 17 5 990 7 9324187

[B29] Gonzalez C Rubio M Romero-Vivas J Gonzalez M Picazo JJ Bacteremic pneumonia due to *Staphylococcus aureus*: A comparison of disease caused by methicillin-resistant and methicillin-susceptible organisms Clin Infect Dis 1999 29 5 1171 7 10.1086/313440 10524959

[B30] Fortun J Navas E Martinez-Beltran J Perez-Molina J Martin-Davila P Guerrero A Short-course therapy for right-side endocarditis due to *Staphylococcus aureus *in drug abusers: Cloxacillin versus glycopeptides in combination with gentamicin Clin Infect Dis 2001 33 1 120 5 10.1086/320869 11389505

